# Supplementary material for: Sex-Specific Anxiety and Prefrontal Cortex Glutamatergic Dysregulation Are Long-Term Consequences of Pre-and Postnatal Exposure to Hypercaloric Diet in a Rat Model
Source: Nutrients. 2020 Jun 19;12(6):1829. doi: 10.3390/nu12061829 (PMC7353464; doi:10.3390/nu12061829)
Supplement: Supplementary file 1 [file nutrients-12-01829-s001.pdf]

Figure S1

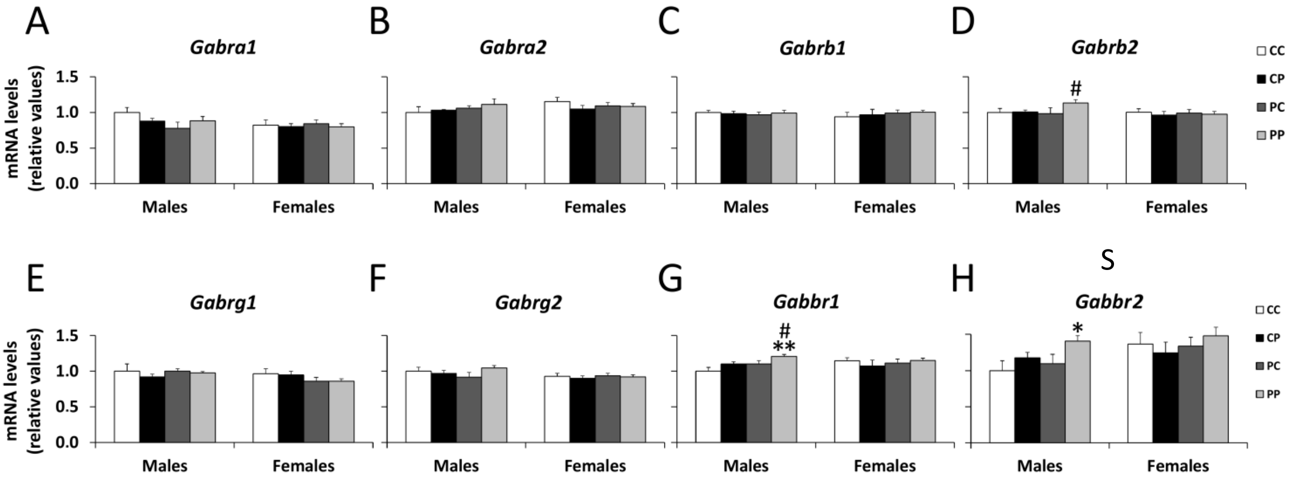

Figure S1. Effect of maternal and/or offspring exposure to a free-choice palatable (P) diet on the relative mRNA levels of GABAergic signaling genes: *Gabra1* (**A**), *Gabra2* (**B**), *Gabrb1* (**C**), *Gabrb2* (**D**), *Gabrg1* (**E**), *Gabrg2* (**F**), *Gabbr1* (**G**) and *Gabbr2* (**H**) in the prefrontal cortex of male and female offspring in adulthood. Data are expressed as the mean ± S.E.M. ($n = 6$). Student's t test: $^{*/**}$ $p < 0.05/0.01$ vs. CC males or CC females; $^{\#}$ $p < 0.05$ vs. CP males or CP females. S: sex effect.

Figure S2

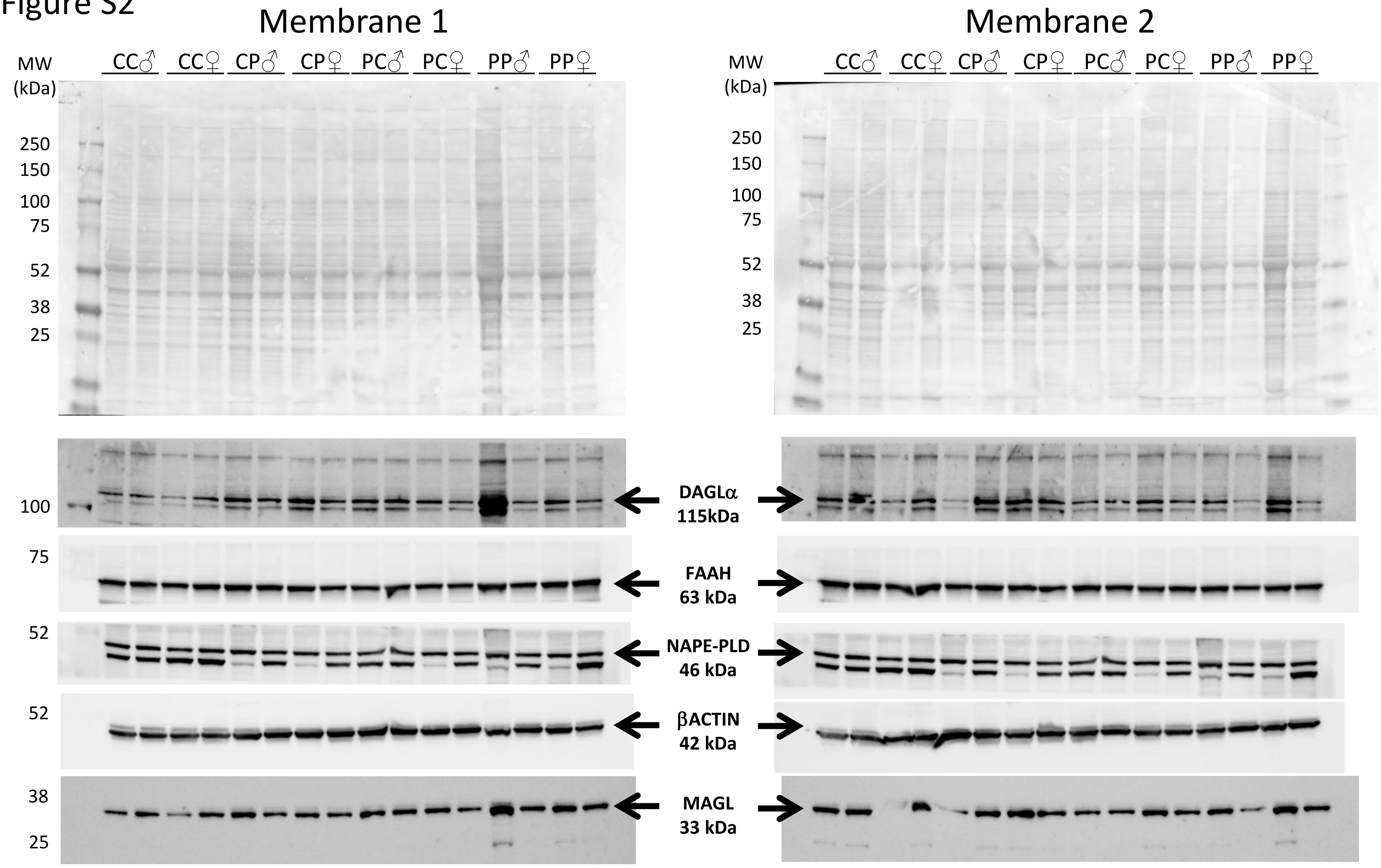

Membrane 1
Membrane 2
MW (kDa)
CC♂ CC♀ CP♂ CP♀ PC♂ PC♀ PP♂ PP♀
250
150
100
75
52
38
25
DAGLα 115kDa
FAAH 63 kDa
NAPE-PLD 46 kDa
βACTIN 42 kDa
MAGL 33 kDa

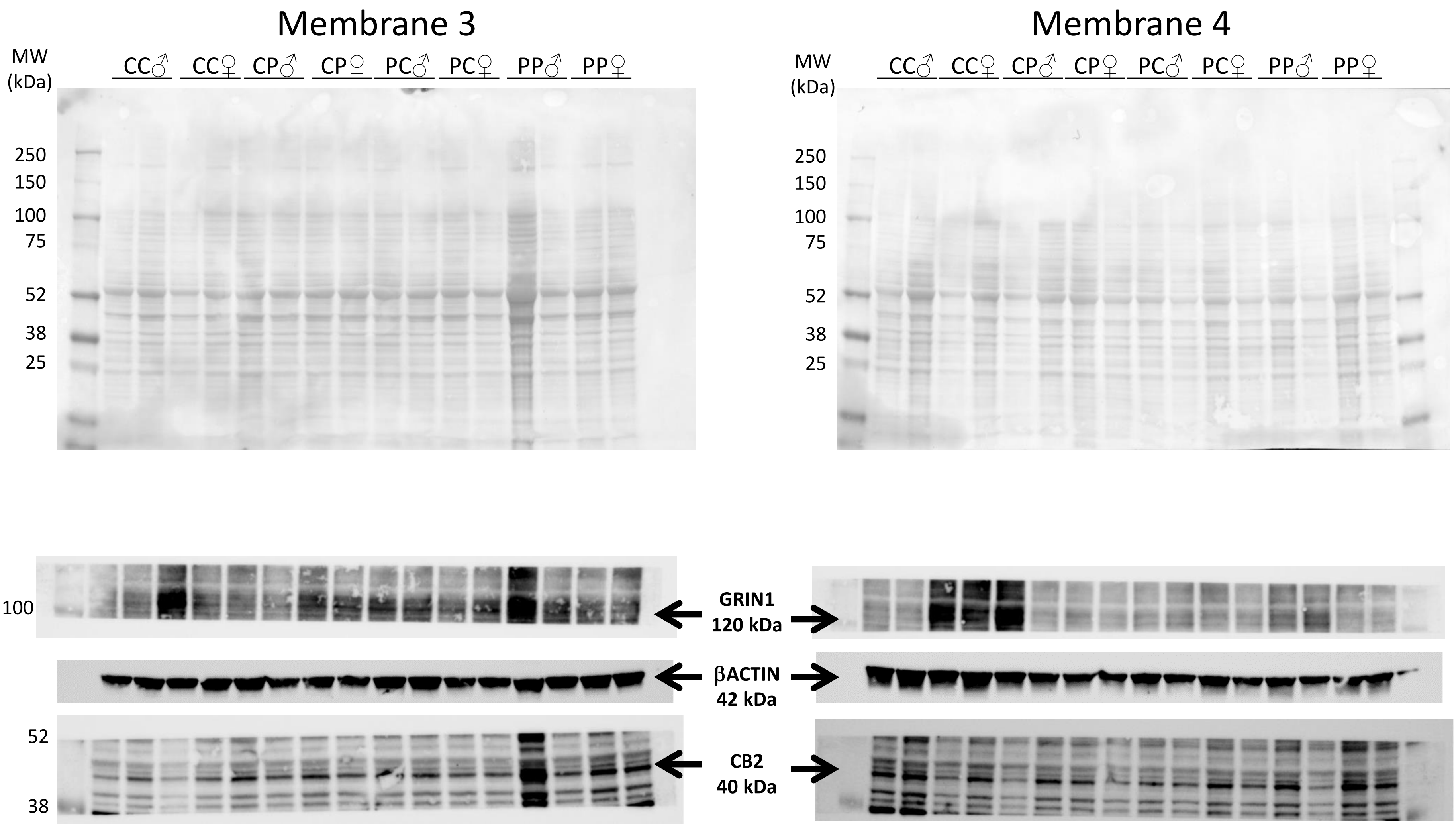

Membrane 3
MW (kDa)
CC♂ CC♀ CP♂ CP♀ PC♂ PC♀ PP♂ PP♀
250
150
100
75
52
38
25
Membrane 4
MW (kDa)
CC♂ CC♀ CP♂ CP♀ PC♂ PC♀ PP♂ PP♀
250
150
100
75
52
38
25
100
GRIN1
120 kDa
βACTIN
42 kDa
52
38
CB2
40 kDa

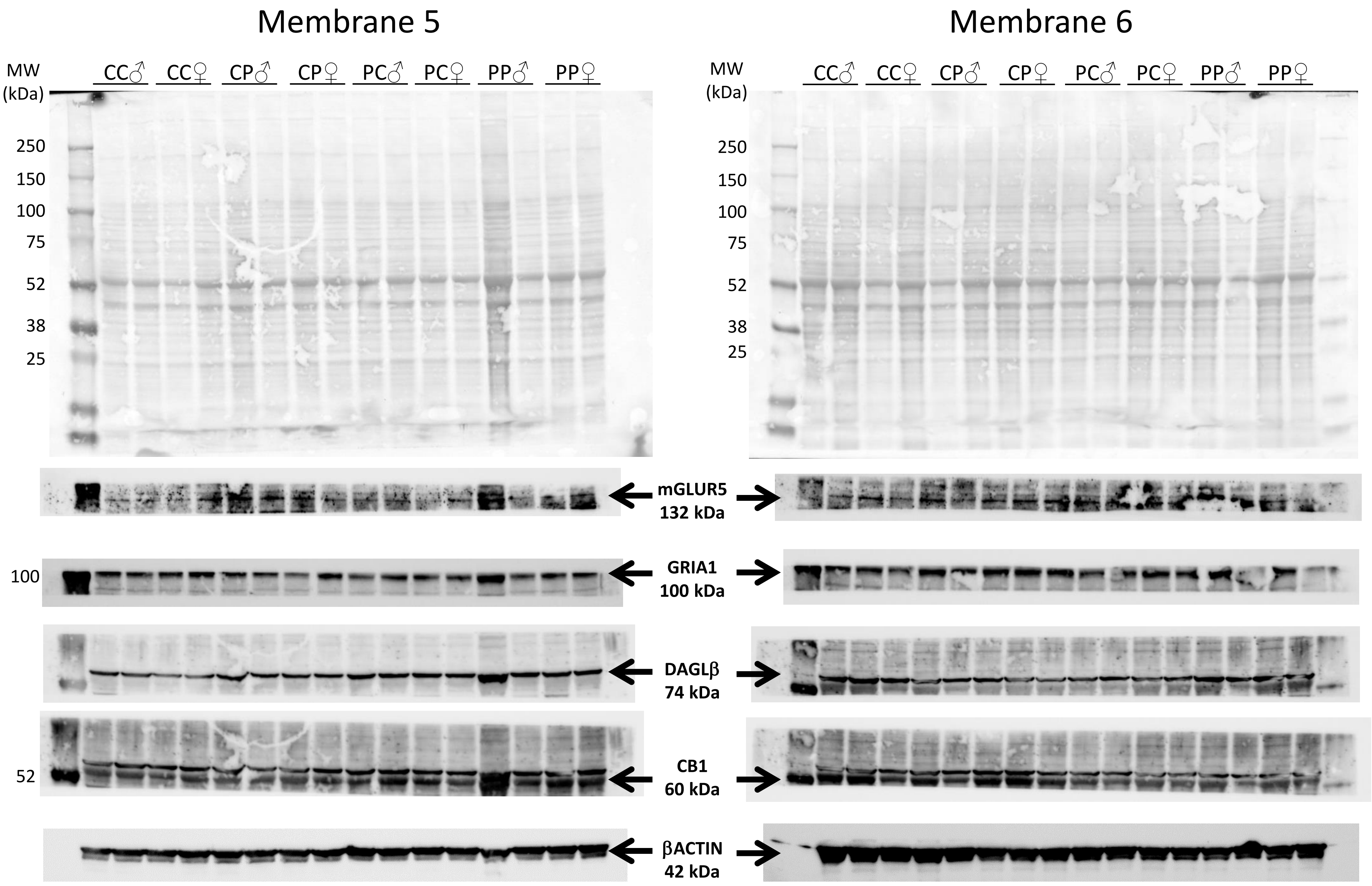
Membrane 5
Membrane 6
MW (kDa)
CC♂ CC♀ CP♂ CP♀ PC♂ PC♀ PP♂ PP♀
250
150
100
75
52
38
25
mGLUR5
132 kDa
GRIA1
100 kDa
100
DAGLβ
74 kDa
CB1
60 kDa
52
βACTIN
42 kDa
